# Advances in Human Dendritic Cell-Based Immunotherapy Against Gastrointestinal Cancer

**DOI:** 10.3389/fimmu.2022.887189

**Published:** 2022-05-10

**Authors:** Ling Ni

**Affiliations:** Institute for Immunology and School of Medicine, Tsinghua University, Beijing, China

**Keywords:** dendritic cells, gastrointestinal cancers, vaccines, immunotherapy, patients

## Abstract

Dendritic cells (DCs), the strongest antigen-presenting cells, are a focus for orchestrating the immune system in the fight against cancer. Basic scientific investigations elucidating the cellular biology of the DCs have resulted in new strategies in this fight, including cancer vaccinology, combination therapy, and adoptive cellular therapy. Although immunotherapy is currently becoming an unprecedented bench-to-bedside success, the overall response rate to the current immunotherapy in patients with gastrointestinal (GI) cancers is pretty low. Here, we have carried out a literature search of the studies of DCs in the treatment of GI cancer patients. We provide the advances in DC-based immunotherapy and highlight the clinical trials that indicate the therapeutic efficacies and toxicities related with each vaccine. Moreover, we also offer the yet-to-be-addressed questions about DC-based immunotherapy. This study focuses predominantly on the data derived from human studies to help understand the involvement of DCs in patients with GI cancers.

## Introduction

In the past decade, the immunotherapy that enhances anti-tumor immunity has revolutionized cancer treatment, leading to potent and durable immune responses in subsets of patients across various tumor types. Immune checkpoint inhibitors (ICIs) that target the T-cell inhibitory checkpoint proteins CTLA-4, PD-1, or the PD-1 ligand PD-L1 have been approved for the treatment of a variety of cancers, including melanoma, non-small-cell lung cancer, head–neck cancer, bladder cancer, renal cell cancer, hepatocellular carcinoma (HCC), and several other tumor types. Despite the huge success these inhibitors have made, only a small subset of cancer patients benefits from ICI therapy. Adoptive chimeric antigen receptor (CAR)-T-cell transfer has also been approved for the treatment of hematological cancers, showing less effectivity against solid tumors.

Gastrointestinal (GI) malignancies are one of the deadliest cancers worldwide. GI malignancies include cancers arising in the oral cavity, esophagus, stomach, liver, pancreas, intestines, rectum, and anus. The GI cancers have a poor prognosis, which mainly depends on tumor stage when diagnosed. Current treatment strategies consist of surgery, chemotherapy, radiotherapy, and targeted therapies. A benefit has been demonstrated in ICI therapy, particularly in esophageal cancer (EC), gastric cancer (GC), and microsatellite instability-high (MSI-H) colorectal cancer (CRC), while pancreatic cancer (PC) and HCC show little response to immune modulation.

Dendritic cells (DCs) play a critical role in the generation of anti-tumor immune responses, mainly acting as the strongest antigen-presenting cells to prime naïve T cells and educating them into cytotoxic T lymphocytes (CTLs). DCs induce an immune response to pathogens and tumors while simultaneously maintaining tolerance to self. DC functional defects have been associated to the progression of various human cancers. Effective cancer vaccines have been a challenge since they target tumor antigens, some of which are self-antigens and thus induce self-tolerance. Here, we performed an extensive literature search of the studies of DC-based immunotherapy against GI cancer patients and provided the advances in DC-based immunotherapy in order to help better understand the status of DC-based immunotherapy.

## Advances in Dendritic Cell-Based Immunotherapy in Esophageal Cancer

EC is a malignancy derived from esophagus, which is the most common digestive tract cancer in China. Despite progresses in various treatment strategies for EC, its 5-year survival rate is approximately 14%–22% with a poor prognosis. Among the various therapeutic strategies, attention has switched to immunotherapy, especially DC-based immunotherapy.

### Dendritic Cells in Esophageal Cancer

For EC patients, there was an increase in the density of S100^+^ DCs in both the tumor stroma and cancer epithelium (1). CD1a^+^ immature DCs (iDCs) were predominantly present in the tumor bed, while DC-Lamp^+^ mature DCs were observed to be exclusive in the tumor stroma. Notably, the density of mature DCs in the tumor mass was greatly reduced compared to that of CD1a^+^ iDCs. The findings indicated that EC tissue comprised a high density of iDCs in the tumor bed and a low density of mature DCs in the tumor stroma (1). Nishimura et al. also evaluated 80 EC patients who underwent surgery without preoperative treatment and found that DC-Lamp^+^ DCs are predominantly located in the peritumor (2). Tumor-infiltrating CD8^+^ T cells (CD8^+^ TILs) were found to be related with a favorable prognosis, and the density of DC-Lamp^+^ DCs was associated with the density of CD8^+^ TILs (2). Although the above advance has been made, the subsets of those intratumoral DCs in EC should be assessed.

### Advances in Dendritic Cell-Based Esophageal Cancer Vaccines

DC-based vaccines are considered an alternative therapeutic approach to treat EC. For EC vaccines, the sources of tumor- associated antigens (TAAs) consisted of the tumor cell lysate, 18E7, and melanoma-associated antigen 3 (MAGE-A3). Gholamin et al. used monocyte-derived DCs to generate EC vaccine (3). In their study, the DCs (culture of monocytes with GM-CSF and IL-4) were transfected with total tumor RNA or normal RNA. Then, T cells were co-cultured with tumor RNA-transfected DCs and normal RNA- transfected DCs, respectively. Only DCs loaded with tumor RNA resulted in the induction of T-cell cytotoxicity and IFN-γ production. These findings offer important preliminary information to develop a total tumor RNA-loaded DC vaccine for EC treatment (3). Wu et al. also generated a DC-based vaccine by HPV18E7 gene-pulsing cord blood CD34^+^ stem cell-derived DC, since human papillomavirus (HPV)-associated EC remains a malignancy with high incidence worldwide (4). They found that HPV18E7 gene transfection did not alter the morphology and phenotypes of mature DCs, while HPV18E7-DCs expressed moderate 18E7 protein. Importantly, the specific T cytotoxicity was significantly higher than that in controls, indicating the possibility of a DC-based vaccine therapy in HPV-associated EC. MAGE-A3 is a tumor-associated antigen target for the generation of anti-tumor DC vaccines against EC. Calreticulin (CALR) is a ligand for nascent major histocompatibility (MHC) class I and supports the induction of DC maturation. Adenovirus (Ad)-transduced DCs that overexpressed MAGE-A3 and CALR demonstrated mature phenotypes (5). Furthermore, the DCs also produced a higher amount of IL-12 and a lower level of IL-10. CALR/MAGE-A3-pulsing DCs activated CD8^+^ CTLs, which killed MAGE-A3-positive EC cells. These data indicated the potential of CALR/MAGE-A3-pulsing DCs to induce MAGE-A3-specific anti-tumor immune responses in EC (5). However, the identification of novel tumor-associated/specific antigens to develop DC-based EC vaccines is imperative.

### Advances in Dendritic Cell-Based Therapy in Esophageal Cancer

Interestingly, the combination therapy of pemetrexed and monocyte-derived DCs (GM-CSF plus IL-4) in the treatment of EC patients who previously failed first-line and second-line treatment regimens could lead to a partial response in a clinical study (6). The patients did not show grade 4 toxicity. The combination therapy of pemetrexed with DCs as a third-line treatment was effective as well as well tolerated in advanced EC patients (6). Immunotherapy using cytokine-induced killer (CIK) cells or a combination of DCs and CIK cells (DC-CIK cells) showed promising clinical outcomes for treating EC. Of note, several groups systematically evaluated the safety and efficacy of CIK/DC-CIK treatment as an adjuvant therapy in the treatment of EC. These meta-analyses indicated that the combination of CIK/DC-CIK immunotherapy with chemotherapy was safe, prolonged the survival time, enhanced immune responses, and improved the treatment efficacy for EC (7, 8).

## Advances in Dendritic Cell-Based Immunotherapy in Gastric Cancer

GC remains the fifth most common cancer and the third deadliest cancer worldwide. Chemotherapy, surgery, and radiotherapy are treatment regimens for GC. However, its 5-year survival rate is 20%–30%. Recently, more and more attention is paid to DC-based immunotherapy.

### Dendritic Cells in Gastric Cancer

The infiltration of CD83^+^ DCs (mature DCs) into the tumors are related with GC development. By the analysis of 55 patients with GC, cytoplasmic TGF-β1 expression was observed in tumor cells from 76.4% of cases, and low CD83^+^ DCs in the tumor border was found in 100% of tumors with TGF-β1 expression (9). TGF-β1 expression was associated with low CD83^+^ DCs in 100% of the cases. The density of CD1a^+^ and CD83^+^ intratumoral DCs was negatively associated with lymph node metastases. Patients with a low density of tumor-infiltrating CD83^+^ DCs had shorter survival rates, Therefore, tumor-infiltrating DCs might be important in initiating the primary anti-tumor immune response. In patients with resectable GC, the density of intratumoral DCs and TGF-β1 expression could be a useful predictor of prognosis (9). Kashimura et al. found a decrease in the number of CD83^+^ DCs and an increase in the density of Foxp3^+^ regulatory T cells (Tregs) in the primary tumor and metastatic lymph nodes (10). Patients with a low number of mature DCs and a high number of Tregs in primary lesions showed a poor prognosis. The number of CD83^+^ mature DCs in negative lymph nodes was an independent predictor of prognosis in patients with metastatic lymph nodes (10).

Both plasmacytoid DCs (pDCs) and Tregs are immunosuppressive cells in the tumor microenvironment (TME) of GC. By the analysis of a cohort of 64 GC patients without preoperative chemotherapy, BDCA2^+^ pDCs and Foxp3^+^ Tregs were increased in tumors and peritumors compared to normal ones, and BDCA2^+^ pDCs were positively associated with Foxp3^+^ Tregs (11). Gastric microbiota dysbiosis might be involved for GC occurrence and progression. The composition, diversity, and function of gastric mucosal microbiota had more significant changes in tumors than those in normal and peritumoral tissues. Several non-abundant genera such as *Selenomonas* and *Stenotrophomonas* were positively associated with pDCs and Tregs, respectively, whereas *Gaiella* and *Comamonas* were inversely associated with pDCs and Tregs, respectively. Gastric mucosal microbiota might regulate the frequency of pDCs and Tregs, which might provide insights into establishing new approaches targeting gastric microbiota (11). Liu et al. also found that both ICOS^+^ Foxp3^+^ Treg cells and pDCs in peripheral blood and tumor tissues could predict poor clinical outcome in GC patients (12). Tregs and ICOS^+^ Tregs are located mainly in tumor tissue, whereas pDCs are present in peritumoral tissue (13). Huang et al. also confirmed that pDCs were positively associated with ICOS^+^ Tregs in peripheral blood and peritumoral tissue from GC patients (13). All these implied that pDCs might get involved in recruiting ICOS^+^ Tregs *via* the ICOS-L/ICOS pathway and both contributed to the immunosuppression in the GC TME.

In the peripheral blood, patients with GC were identified to have a substantially higher percentage of peripheral pDCs and CD1c^+^ myeloid DCs (mDC2) (14). Moreover, there was a trend of elevated circulating pDCs toward advanced stages and lymph node metastasis, while there were no differences in blood mDC2s among the various groups, suggesting that blood pDCs were a prognostic factor in GC patients and emphasizing the pivotal role of pDCs in the progression of GC (14). The phenotype and function of mDCs in the tumor mass from GC patients warrant further investigation.

### Advances in Dendritic Cell-Based Gastric Cancer Vaccines

Cancer stem cells (CSCs) and GC cell lines were used for antigen targets for DC-based vaccines. CSCs are a small subset of cancer cells in solid tumors, which participate in tumor initiation, progression, recurrence, metastasis, and resistance to current treatments. Bagheri et al. generated DCs by culturing monocytes with GM-CSF plus IL-4 (GM/IL-4 DCs) while maturing them by the cytokine mixture (TNF-α, IL-1β, IL-6, and PEG2). Matured DCs pulsed with CSC mRNA induced Ifng gene expression in T cells (15). The cytotoxic activity of primed T cells with CSC antigens was significantly enhanced compared to control groups. Thus, the DCs loaded with CSC mRNA that elicited tumor-specific T-cell immune responses might be a potential DC-based vaccine for GC patients (15).

The cell lysate antigen of SGC-7901 cells is another option for DC-based GC vaccines. The secondary lymphoid tissue chemokine (SLC) is a chemokine in the T-cell zones of the spleen and lymph nodes as well as in endothelial venules. Human GM/IL-4 DCs were transduced with Ad-bearing SLC (Ad-SLC) and the recombinant Ad bearing the beta-galactosidase gene, respectively (16). Then, the DCs were loaded with the cell lysate antigens of SGC-7901 cells and co-cultured with autologous T cells. Transduction with Ad-SLC led to DC maturation and enhanced the chemotaxis function of DCs. Moreover, Ad-SLC transduction also led to upregulated RANTES expression and a similar level of IL-10 and IL-12p70 in DCs. The co-culture of autologous T cells with SGC-7901 cell lysate-loaded SLC^+^ DCs led to a significantly promotion in the proliferation of autologous T cells and a strong cytotoxicity against SGC-7901 cells. Collectively, Ad-SLC enhanced DC maturation and, in turn, increased T-cell chemotaxis and elicited a specific GC-specific immune response. Thus, recombinant Ad-SLC-transduced DCs might be exploited as an adjuvant to induce an effective anti-GC cellular immunity (16).

### Advances in Dendritic Cell-Based Therapy in Gastric Cancer

Cord blood-derived DCs and CIK (CB-DC-CIK) were clinically exploited for the treatment of GC (17). Patients with advanced GC were treated with CB-DC-CIK plus chemotherapy. No serious adverse effects were observed in patients with GC after the application of CB-DC-CIK. The combination therapy led to a significant increase in the overall disease-free survival (DFS) rate in comparison to chemotherapy alone. Furthermore, the percentage of CD4^+^ T cells, NK cells, and NKT cells and the levels of IFN-γ, TNF-α, and IL-2 were significantly increased in the experimental group. Thus, the combination therapy of CB-DC-CIK and chemotherapy was safe and effective for the treatment of patients with advanced GC (17).

## Advances in Dendritic Cell-Based Immunotherapy in Hepatocellular Carcinoma

HCC is one of the most common cancers worldwide with limited therapeutic strategies due to HCC-induced immunosuppression. The 5-year survival rate is less than 20%. Recently, a growing body of studies supported that the function of DCs in HCC was impaired.

### Dendritic Cells in Hepatocellular Carcinoma

Huang et al. performed the weighted gene co-expression network analysis of DCs in HCC patients in public datasets (18). They observed that a high level of DC infiltration was correlated with poor prognosis. By the analysis of a TCGA cohort, more than 50% of the DC-related genes were markedly differentially expressed between HCC and normal samples. There were 17 differentially expressed genes (DEGs) significantly related with overall survival (OS) (18), implying that intratumoral DC might get involved in HCC progression.

Human CD14^+^ CTLA-4^+^ regulatory DCs (CD14^+^ DCs) were identified, which accounted for approximately 13% of peripheral blood mononuclear cells (PBMCs) (19). CD14^+^ DCs significantly suppressed the T-cell response *in vitro via* indoleamine-2,3-dioxygenase (IDO) and IL-10, which also expressed high levels of CTLA-4 and PD-1. CTLA-4 was essential for IL-10 and IDO production. These data indicated one underlying mechanism by which CD14^+^ DCs elicited systemic immunosuppression in HCC, participating in HCC progression. This might also offer a previously unrecognized target of HCC immunotherapy (19). PD-1 expressed on DCs has a regulatory role in the anti-tumor immune response. Lim et al. observed PD-1 expression on all DC subsets (CD1c^+^ mDC2, CD141^+^ mDC1, and pDCs) in the peripheral blood of HCC patients (20). However, PD-1 was weakly expressed in mDC1 but not mDC2 and pDCs in the steady state. This finding provided a new insight into the mechanisms for PD-1-targeted cancer immunotherapies.

The number of tumor-infiltrating Tr1 cells (CD4^+^ FoxP3^-^ IL-13^-^ IL-10^+^) was associated with tumor-infiltrating pDCs, which enhanced Tr1-produced IL-10 *via* ICOS-L when exposed to tumor-derived factors. The Tr1 cells were characterized in the tumors from individuals with HCC or liver metastases from CRC, which expressed CD49b and the lymphocyte activation gene 3 (LAG-3). Moreover, Tr1 had a strong suppressive activity of T-cell responses in an IL-10-dependent way (21). These findings suggested a role of pDC-expressing ICOS-L in enhancing intratumoral Tr1-mediated immunosuppression in human HCC (21).

Some factors in the TME could modify the function of tumor-infiltrating DCs, such as the liver X receptor (LXR), IL-37 and alpha-fetoprotein (AFP). Sterol metabolism is linked to innate and adaptive immunity *via* LXR signaling. Human tumors express LXR ligands that reduced CCR7 expression on mature DCs and, in turn, their migration to lymphoid organs. CD83^+^CCR7^-^ DCs within the human HCC tumor could be detected (22). Thus, these findings indicated a new mechanism of tumor immune escape involving the products of cholesterol metabolism (22). Targeting this pathway could restore anti-tumor immunity in individuals with HCC. In GC, SLC could activate DCs by upregulating CCR7 and CD83 expression. The function of SLC should be addressed in the context of HCC. IL-37 is a tumor suppressor in various cancers. The amount of IL-37 was positively correlated with CD1a^+^ iDC infiltration in HCC specimens (23). The survival rates of patients with both a high amount of IL-37 and a high number of iDCs were significantly enhanced compared with those of patients with low levels of IL-37 and iDCs. HCC cells that overexpressed IL-37 recruited more DCs through secreting specific chemokines. Moreover, IL-37 indirectly upregulated the expressions of MHC class II molecules, CD86 and CD40, on DCs and, in turn, increased T-cell-mediated anti-tumor immunity by inducing DCs to producing cytokines. Thus, DCs were responsible for IL-37-induced anti-tumor immunity in HCC, which might help develop novel cancer immunotherapeutic approaches (23). AFP is an oncofetal antigen and considered as the most common serum biomarker. HCC patients with high amounts of serum AFP showed a lower ratio of myeloid/plasmacytoid DCs in comparison to patients with low serum AFP as well as healthy donors (24). Although the isoforms of cord blood-derived normal AFP (nAFP) and HCC tumor-derived AFP (tAFP) only vary at one carbohydrate group, low amounts of tAFP, but not nAFP, markedly suppressed monocyte-derived DC differentiation. Importantly, tAFP-educated DCs expressed reduced levels of DC maturation markers and maintained a monocyte-like morphology and therefore failed to elicit potent T-cell proliferative responses. Collectively, novel immunotherapeutic strategies that target tAFP might be crucial to improve immune responses and clinical outcomes (24).

### Advances in Dendritic Cell-Based Hepatocellular Carcinoma Vaccines

AFP is a promising tumor-associated antigen target for the generation of DC-based vaccines. An AFP-derived peptide-loaded DC vaccine could promote an AFP-specific anti-tumor immune response in patients with HCC, and the clinical trial results showed these vaccine-induced CD8^+^ T-cell responses (25). In addition, AFP-derived peptide-pulsed DCs enhanced NK cell activation and decreased the frequency of Treg cells in vaccinated HCC patients. Various antigen-loading approaches are related with the efficacy of DC-based vaccines. The recombinant adeno-associated virus (rAAV) is one safe virus vector in gene therapy, since the wild-type virus does not cause human disease. The study by Zhou et al. supported the superiority of the rAAV-AFP-engineered DC vaccine over the cancer cell lysate-loaded DC vaccine (26). Both rAAV-AFP-loaded and cancer cell lysate-loaded DCs led to DC maturation. However, rAAV-AFP-loaded DCs induced T-cell responses more potent than cancer cell lysate- pulsed DCs. Thus, the DCs loaded with rAAV-AFP were more effective than the DCs loaded with the tumor cell lysate, which might be exploited for the development of DC-based vaccines in AFP^-^positive HCC (26).

Heat shock protein 70 (HSP70) is highly expressed in HCC, being one of HCC-associated antigens. A clinical phase I/II trial investigated the safety and efficacy of HSP70 mRNA-pulsing DC vaccine that was as a postoperative adjuvant therapy after tumor resection. There were no side events specific to the mRNA-pulsing DC vaccine. Similar DFS between the DC and control groups was observed. However, the OS of the DC group was significantly prolonged compared to that of the control group. Collectively, HSP70 mRNA-pulsing DC vaccines were safe as an adjuvant therapy (27).

Exosome is a subtype of membrane vesicle released from the cells or directly from the plasma membrane. DC-derived exosomes (DEXs) become a new class of vaccines for cancer immunotherapy and thus provide a cell-free vaccine for HCC immunotherapy. Li et al. found that GM/IL-4 DCs were loaded with recombinant rAAV/AFP and high-purity DEXs were generated. DEXs were found to be effective at inducing antigen-specific CTLs, demonstrating anti-tumor immunity against HCC (28). Moreover, DEX-sensitized DC precursors were likely to be more effective at triggering an MHC class I-restricted CTL response, allowing DCs to make full use of the minor antigen peptides and, in turn, maximally promoting specific immune responses against HCC. Thus, DEXs might replace mature DCs to act as cancer vaccines (28).

The tumor cell line lysate provides whole tumor antigens for the generation of HCC vaccines. Thirty HCC patients were divided into 2 groups in a clinical study. Group 1 (15 patients) received intradermal vaccination with mature DCs loaded with a liver tumor cell line lysate, while group 2 received supportive treatment (29). The patients in group 1 showed enhanced CD8^+^ T-cell responses and improved OS. Thus, DC vaccination in advanced HCC patients was safe and well tolerated (29). However, the efficacy of this approach is not most satisfactory. Identifying new HCC-associated/specific antigens is quite urgent for developing DC-based HCC vaccines.

Another clinical trial investigated the safety and efficacy of the combination therapy of an autologous tumor lysate-loaded DC vaccine with *ex vivo* activated T-cell transfer (ATVAC) in a postoperative adjuvant therapy (30). Ninety-four patients with invasive HCC were enrolled, and 42 had the ATVAC after surgery. Compared with surgery alone, surgery plus ATVAC significantly enhanced the recurrence-free survival (RFS) and OS. There were no adverse events of grade 3 or more. Thus, the combination therapy of postoperative DC vaccine with T-cell adoptive transfer might be an effective and feasible treatment for preventing recurrence in HCC patients (30).

Although PD-1 blockade therapy got many successes and opportunities in various cancers, anti-PD-1 monoclonal antibodies still confronted several challenges. Shi et al. generated a nanobody (Nb) against PD-1 (PD-1 Nb20) to solve these challenges (31). The combination treatment of PD-1 Nb20 with the tumor-specific DC/tumor-fusion cell (FC) vaccine was observed to effectively improve the *in vitro* cytotoxicity of CD8^+^ T cells to kill HCC HepG2 cells. In addition, the combination therapy was approved to be potent in a mouse tumor model. Collectively, these findings indicated that the combination therapy of PD-1 Nb20 with DC/tumor-FC vaccines greatly improved CTL capacity, offering a promising strategy for tumor patients who were not sensitive to anti-PD-1 therapy (31).

### Advances in Dendritic Cell-Based Therapy in Hepatocellular Carcinoma

DC-CIK immunotherapy is also found to be safe and effective in the treatment of HCC patients. In a clinical cohort of 67 HCC patients treated with DC-CIKs (32), 29 patients displayed stable disease (SD) with none showing complete remission and five undergoing partial remission. DC-CIK cells had a great effect on the growth cycle of HepG 2 cells, mainly upregulating the gene expression of BAX (a pro-apoptotic protein) and downregulating the activity of proliferating cell nuclear antigen (PCNA, marker of the cell proliferation status). Thus, the co-culture of DCs and CIK cells inhibited the proliferation and migration of HCC cells by the regulation of PCNA and BAX. This strategy might be an effective method to treat advanced HCC (32). Yang et al. also found that DC-CIKs significantly enhanced the apoptosis ratio by increasing caspase-3 protein expression and reducing PCNA expression against liver cancer stem cells (LCSCs) (33). Su et al. showed that the combination of DC-CIK immunotherapy and transcatheter arterial chemoembolization (TACE) or TACE plus local ablation therapy improved 1- and 2-year OS and provides a better quality of life for patients with HCC clinically (34).

OK-432, a streptococcus-derived tumor suppressor, can activate DCs and, in turn, improve anti-tumor activity. In a clinical study, GM/IL-4 DCs were launched and stimulated with OK-432 (35). Two groups of HCC patients were treated with transcatheter hepatic arterial embolization (TAE) alone and TAE plus OK-432-matured DC transfer, respectively. OK-432 induced DC maturation, which expressed high levels of a homing receptor, preserved phagocytic capacity and markedly improved cytokine production as well as tumoricidal activity. The infusion of OK-432-matured DCs to HCC patients was safe and feasible. In addition, the combination therapy of TAE with OK-432-activated DCs prolonged the RFS of patients compared with the TAE alone group. Thus, these findings indicated that the combination therapy of a DC-based immunotherapeutic approach with locoregional treatments benefited HCC patients (35).

The efficacy of the combination therapy of DC-CIKs pretreated with pembrolizumab (anti-PD-1) against HCC was also clinically investigated (36). The PD-1 blockade further improved the anti-tumoral effects of DC-CIKs, leading to a survival benefit. The blockade of PD-1 promoted the infiltration of immune cells into tumors. DC-CIKs and anti-PD-1 were more effective than DC-CIKs alone (36). Thus, blocking the PD-1/PD-L1 signaling pathway in DC-CIK cells prior to infusion is a promising treatment strategy against HCC.

## Advances in Dendritic Cell-Based Immunotherapy in Pancreatic Cancer

PC is a challenging disease with a high mortality rate that might be associated with defective immune function. It has been reported that the function of DCs is impaired in PC patients. More and more evidence supported that blood mDCs and pDCs in PC showed decreased numbers and impaired functionality.

### Dendritic Cells in Pancreatic Cancer

Pancreatic ductal adenocarcinoma (PDAC), the most common PC, is recognized to be a very aggressive tumor type with very high mortality. PDAC had decreased levels of blood mDCs and pDCs and an enhanced apoptosis of these DCs (37). Enhanced levels of PGE2 and CXCL8 were observed in subjects with PDAC and chronic pancreatitis. After tumor resection, the amounts of these inflammatory factors were partially recovered in PDAC, while the percentages of DCs were impaired in most of these patients approximately 12 weeks after tumor resection. These findings proved that solid PC, including PDAC, systemically altered blood DCs. The impaired DCs might not be tumor specific since chronic pancreatitis could also lead to similar results (37). Moreover, PDAC patients with long survival had significantly increased frequency of blood DCs in comparison to patients with short survival. Thus, inflammation contributed to the impairment of the blood mDCs and pDCs, while the preservation of the blood DCs might control the disease in PDAC patients (37). Furthermore, the blood DCs showed a partial maturation phenotype with a significantly increased expression of CD40, CD83, B7-H3, PD-L1, CCR6, and CCR7 and a decreased expression of DCIR and ICOSL in PDAC patients, which were partially induced by PGE2 (38). The alternations led to an impaired function of DCs. However, chronic pancreatitis also led to a similar partial mature phenotype of DCs as in PDAC. Thus, it was the systemic inflammation that contributed to semi-mature DCs in PDAC patients *via* PGE2. Diminishing the inflammation to preserve functional blood DCs could help control the disease and improve survival (38). A similar finding was observed in the other cohort of patients with PC, in which the frequency of the circulating mDCs in the patients was significantly reduced compared to that in healthy donors. There was no obvious difference in the blood mDCs between the patients with distant organ metastasis and locally advanced PC. The patients with more blood mDCs survived longer than patients with less (39, 40).

The transition of chronic pancreatic fibroinflammatory disease to tumorigenesis is a paradigm linking inflammation to carcinogenesis. The lipopolysaccharide (LPS) treatment facilitated pancreatic tumorigenesis (41). Blocking the MyD88-independent TRIF pathway resulted in protection, while blocking the MyD88-dependent pathway unexpectedly exacerbated pancreatic inflammation and tumor progression (41). Of note, DCs mediated MyD88 suppression, which induced a pancreatic antigen-restricted Th2 cell response and promoted the transition from pancreatitis to neoplasia, indicating that DCs played a critical role in pancreatic carcinogenesis and neoplastic transformation through promoting the DC-Th2 axis (41).

Tumor-derived exosomes (TDXs) can transfer miRNAs to recipient cells in the TME, promoting tumor invasion and metastasis. In context of PC, TDX miRNAs inhibited the mRNA expression of DCs and induced immune tolerance (42). Mechanistically, the miR-212-3p from PC-secreted exosomes downregulated regulatory factor X-associated protein (RFXAP) and, in turn, decreased MHC class II expression. In addition, one clinical study showed that miR-212-3p was inversely correlated with RFXAP in PC tissue. However, the functions and mechanisms of RFXAP in tumors warrant future investigation (42). miRNA-146a can also modulate DCs. The culture of human CD14^+^ monocyte-derived DCs by a highly metastatic human PC cell line BxPC-3 culture media (BxCM) resulted in decreased DC differentiation and antigen- presentation function (43). BxCM-treated DCs upregulated miRNA-146a, the inhibition of which partially rescued the BxCM-induced impairments in DC differentiation and function (43).

In PDAC, Treg cells extensively interacted with tumor-associated mDCs and reduced the expression of costimulatory molecules that was required for the activation of CD8^+^ T cells (44). Resultantly, CD8^+^ TILs showed impaired effector function when Treg cell ablation was combined with DC depletion. These findings indicated that Treg TILs could promote immune tolerance by inhibiting intratumoral DC maturation (44). Trefoil factor 2 (TFF2) from PC cells, a chemokine/cytokine, may attract iDCs and affect the initial stage of DC maturation, thereby contributing to the induction of immune tolerance against PC (45). As a PC-derived factor, regenerating islet-derived protein 3A (Reg3A) could inhibit the differentiation and maturation of tumor-infiltrating DCs *via* the Reg3A-JAK2/STAT3 signaling pathway (46). HSP70 contributed to cell survival and tumor progression. HSP70 inhibition in DCs may emerge as a novel therapeutic strategy against pancreatic cancer (47).

### Advances in Dendritic Cell-Based Pancreatic Cancer Vaccines

Various PC cell lines (Panc-1, KP-1NL, QGP-1, and KP-3L) were used to load DCs to generate DC-based vaccines against PC (48). The cytotoxicity against tumor cells induced by GM/IL-4 DCs fused with QGP-1 (DC/QGP-1) was very low, while DCs loaded with another PC cell line elicited a high cytotoxicity of PBMCs. DC/QGP-1 upregulated Treg expansion in comparison with DC/KP-3L (48). Moreover, the co-culture of DC/QGP-1 with PBMCs also resulted in an increase in the amount of IL-10 compared with that with DC/KP-3L. Thus, the cytotoxicity induced by DCs loaded with PC cell lines was variable among the cell lines. DC/QGP-1-mediated reduced cytotoxicity might be associated with IL-10 production and Treg expansion (48).

Various antigen-pulsing approaches have been investigated (49). Both tumor RNA and tumor fusion hybrid cells provide whole tumor antigens for the generation of DC-based vaccines. Chen et al. compared the anti-tumor immunity elicited by DC-tumor hybrids (patient-derived PC cells and DCs were fused) and DC-tumor RNA (autologous DCs were transfected with primary PC cell-derived total RNA) (49). They found that both RNA transfection and hybrid techniques could elicit tumor-specific CTL responses. However, DCs loaded with total tumor RNA led to an increase in the frequency of activated CTLs and CD4^+^ T cells compared to DC-tumor hybrids. Moreover, DCs pulsed with tumor RNA induced stronger autologous tumor cell lysis. Thus, DC-tumor RNA was superior to DC-tumor hybrids in stimulating PC-specific CTL responses (49). Pancreatic CSCs participated in the malignant behaviors of PC, such as immune escape. Therefore, the development of immunotherapy-targeting pancreatic CSCs might contribute to PC treatment. Yin et al. cultured Panc-1 cells under sphere-forming conditions to enrich pancreatic CSCs (50), which expressed low levels of HLA-ABC and CD86. DCs loaded with Panc-1 CSC lysates elicited cytotoxicity against Panc-1 CSCs and parental Panc-1 cells. Thus, a CSCs-DC-based vaccine might be a promising approach for the treatment of PC (50).

Wilms’ tumor 1 (WT1) has been used to generate a DC-based vaccine against PC. Three types of MHC class I and II-restricted WT1 epitopes have been identified. A clinical phase I trial investigated safety, clinical responses, and WT1-specific T-cell immune responses for DCs loaded with a mixture of three types of WT1 peptides (DC/WT1-I/II), in combination with chemotherapy (51). The combination therapy of DC/WT1-I/II and chemotherapy was well tolerated. In addition, the combination therapy of DC/WT1-I/II resulted in a significant increase in the percentage of WT1-specific IFN-γ-producing CD4^+^ T cells. Among the PDAC patients vaccinated with DC/WT1-I/II, 4 of the 7 patients showed WT1 peptide-specific delayed-type hypersensitivity (DTH). Improved OS and PFS were detected in the WT1-specific DTH-positive patients in comparison with the negative-control patients. More importantly, all three PDAC patients with strong DTH had a median OS of 717 days. Thus, WT1-specific immune responses induced by the combination therapy of DC/WT1-I/II with chemotherapy might be related with disease stability in advanced PC (51). In addition, seven patients with PC who received the combination therapy of DC/WT1-I/II and chemotherapy showed significantly increased expressions of HLA-DR and CD83 on DCs (52). Therefore, the enhanced expressions of HLA-DR and CD83 might be prognostic markers of longer survival in patients with advanced PC who underwent chemoimmunotherapy (52).

Mesothelin (MSLN) is a potential candidate as a molecular target for PC immunotherapy. GM/IL-4 DCs were adenovirally transduced with the full-length MSLN gene (DC-AxCAMSLN) (53). Target cells were PC cell lines (PK1, CfPAC1, AsPC1) transduced with the MSLN gene. DC-AxCAMSLN stimulated MSLN-specific CTLs, which resultantly killed target cells. Both CD8^+^ T cells and CD4^+^ T cells sorted from these CTLs expressed significant levels of IFN-γ. In addition, the DC-AxCAMSLN also elicited a potent MSLN-specific cytotoxic activity against PC cell lines endogenously expressing MSLN (53). Therefore, the findings suggest the potential of developing DC-based vaccines using genetically modified DCs expressing MSLN.

Mucin 1, an epithelial cell glycoprotein, is aberrantly overexpressed in many cancers, including PC, providing an ideal TAA target for immunotherapy. GM/IL-4 DCs were generated and matured with TNF-α. Mature DCs could be transfected with amplified mucin 1 mRNA efficiently (54). The mucin 1-loaded DCs were robustly effective in stimulating mucin 1-specific CTL responses, which could only recognize and kill HLA-A2-restricted mucin 1-expressing PC and other target cells, providing a preclinical rationale for DC-based vaccines using mucin 1 as a target (54). The mucin 1 peptide epitope was identified. In a clinical phase I trial, TNF-α-stimulated GM/IL-4 DCs were pulsed with the mucin 1 peptide epitope (55). Mucin 1-positive patients with recurrent lesions or metastasis after surgery received DC vaccines intradermally for three or four vaccines. The patients did not show severe adverse events related with the vaccines or an autoimmunity symptom. Approximately 2 out of 7 patients expressed IFN-γ and granzyme B. The administration of mucin 1-peptide-pulsed DCs was well tolerated and able to induce a mucin 1-specific immune response in advanced PC patients. Further studies were needed to improve tumor rejection responses (55). Mucin 4 and survivin are another two TAA targets for DC-based vaccines. DCs co-transfected with two mRNAs encoding mucin 4 and survivin induced more potent CTL responses against PC target cells in comparison with the DCs transfected with a single mRNA (56). These findings provided a rationale for the clinical studies of DC vaccines encoding multiple TAA epitopes (56).

pBSDL-J28 is a glycoform of bile salt-dependent lipase (BSDL) in normal human pancreatic juices, which is not expressed in nontumoral pancreatic tissues and cells. Of note, pBSDL-J28 induced DC maturation and these DCs kept their full ability to internalize antigens, making this maturation atypical. In addition, the allogeneic pBSDL-J28-treated DCs induced proliferative responses of lymphocytes. DCs loaded with the pBSDL-J28 C-terminal glycopolypeptide and stimulated with CD40L elicited T-cell proliferation (57). Therefore, pBSDL-J28 might be a promising TAA target for DC-based vaccine against PC.

Three distinct HLA-A2-restricted peptide epitopes were identified: human telomerase reverse transcriptase (hTERT, TERT572Y), survivin (SRV.A2), and carcinoembryonic antigen (CEA; Cap1-6D). Mehrotra et al. used these three peptide-loaded DCs in conjunction with TLR-3 agonist poly-ICLC to treat PC patients with metastatic or locally advanced unresectable PC in a clinical study (58). This treatment was well tolerated. An MHC class I tetramer analysis indicated a potent induction of antigen-specific T cells in three PC patients with SD. Thus, vaccination with peptide-pulsed DCs plus poly-ICLC was safe and elicited detectable tumor specific T-cell responses in patients with advanced PC (58).

Chemotherapy enhances the efficacy of DC-based vaccines for PC. Gemcitabine is a first-line chemotherapeutic drug for advanced PC. The medium of gemcitabine-treated PC cells stimulated DC maturation (59). The co-culture of gemcitabine-treated DCs with autologous T lymphocytes resulted in T-cell proliferative responses and the induction of specific anti-tumor CTLs. Enhanced DC maturation might be associated with the enhanced level of HSP70 by gemcitabine. Thus, gemcitabine changed the immunogenicity of tumor cells and enhanced the efficacy of DC-based vaccines for PC (59). In addition, gemcitabine inhibited the growth of PC cells by inducing apoptosis and upregulating Fas expression. Thus, gemcitabine sensitized PC cells to the CTL antitumor response, which was related with the upregulation of Fas on PC cells (60).

### Advances in Dendritic Cell-Based Therapy in Pancreatic Cancer

DC-CIK immunotherapy has widely used in treating PC patients. Both chemotherapy drugs and miRNA-depleted TDXs could enhance the efficacy of DC-CIK immunotherapy. Zhang et al. conducted a meta-analysis of 14 clinical trials with 1,088 PC patients to compare DC-CIK immunotherapy plus chemotherapy (combined therapy) with chemotherapy alone (61). The combination therapy showed advantages over chemotherapy alone. The percentages of CD3^+^ T cells, CD4^+^ T cells, and CD3^+^CD56^+^ T cells as well as the cytokine levels of IFN-γ were significantly increased by the combined therapy. Moreover, the combination of DC-CIK immunotherapy and chemotherapy enhanced the PC patients’ survival time, being an effective treatment strategy (61). TDXs might be potential candidates for tumor vaccines since they have numerous immune-regulating proteins. TDX-derived miRNAs, however, elicit immune tolerance. Que et al. depleted miRNA from PC-derived exosomes and retained 128 proteins, including several immune-activating proteins (62). Exosomes were depleted with miRNA-activated DC/CIKs and resultantly induced a higher cytotoxic capacity of CIKs than LPS and exosomes. Therefore, an miRNA-depleted exosome could be a promising agonist for stimulating DC/CIKs against PC (62).

The iDCs plus OK-432 in PC patients were administrated by preoperative endoscopic ultrasound-guided fine-needle injection (PEU-FNI) (63). The clinical trial included two groups, the DC group with iDC injection and non-DC group without iDC injection (non-DC group). The patients did not show severe toxicities in the DC group, except for one transient grade 3 fever. A similar incidence of postoperative complications was detected between the two groups (63). In the DC group, more CD83^+^ cells presented in the regional lymph nodes and Foxp3^+^ cells in the regional and distant lymph nodes. The two PC patients from the DC group with one being stage IV survived over 5 years without requiring adjuvant therapy (63). Thus, PEU-FNI was safe and feasible. Further investigation was warranted to confirm and enhance anti-tumor responses.

A direct injection of DCs without loading TAAs into tumors after chemotherapy-mediated apoptosis is more feasible. Zoledronate is the most potent and long-acting bisphosphonate, which is intended for clinical use. Hirooka et al. studied the safety, feasibility, and efficacy of one immunotherapy regimen including the combined intratumoral injection of zoledronate-treated DCs (Zol-DCs), gemcitabine, and T cells in locally advanced PC (64). Approximately 7 out of 15 patients underwent an SD, and the majority of the patients mounted long-term clinical responses. Additionally, the CD8^+^/Treg ratio was significantly increased in SD patients after treatment. Before treatment, the patients with a neutrophil/lymphocyte ratio (NLR) lower than 5.0 underwent significantly longer survival. Thus, the combination therapy of Zol-DCs, systemic T cells, and gemcitabine might show a synergistically therapeutic effect on locally advanced PC. The combined immunotherapy might benefit patients with PC if precise biomarkers were used (64).

## Advances in Dendritic Cell-Based Immunotherapy in Colorectal Cancer

CRC remains the second most deadly cancer in Western countries. Chronic inflammation is a key component in the development and progression of CRC. Defects in DC recruitment, maturation, and cytokine release are a hallmark of the CRC strategy to escape immune surveillance.

### Dendritic Cells in Colorectal Cancer

The analysis of a cohort of CRC patients revealed a high CD1a^+^/DC-LAMP^+^ tumor-infiltrating DC ratio, indicating that there were more iDCs than mature DCs in the CRC tumors (65). Moreover, there were decreased mature DCs in the front and main tumor mass (66), while increased mature DCs in both of these locations were correlated with the metastases in the nearby lymph nodes. Of note, increased amounts of mature DCs in the tumor was correlated with the invasiveness of the tumor and especially with the metastasis to the surrounding lymph nodes (66). Gai et al. also showed that increased FOXP3^+^ Tregs and decreased CD11c^+^ mDC infiltration had a strong prognostic significance in CRC (67). Unlike FOXP3^+^ Tregs, the frequency of CD123^+^ pDCs was lower in most CRC tumor tissues (68).

In blood, the absolute number of pDCs in CRC patients with stage III–IV patients was significantly reduced compared with controls at the pre-operative time point, while the number of mDCs in CRC patients did not show an obvious difference in comparison to that in controls (69). Interestingly, the tolerogenic antigen CD85k was expressed highly on mDCs in CRC patients. CRC cells expressed anti-inflammatory cytokines such as IL-10 and TGF-β that could modulate the DC phenotype and supported tumor escape. Resultantly, tumor-infiltrating DCs displayed impaired antigen-presenting capacity and altered the expression pattern of immune costimulatory molecules (70). Thus, these alterations seemed to be correlated to cancer progression. This knowledge might contribute to the development of more efficacious immunotherapeutic approaches (69).

### Advances in Dendritic Cell-Based Colorectal Cancer Vaccines

The tumor cell line lysate is also source of TAAs that are loaded on DCs. Chen et al. generated GM/IL4DCs, which were pulsed with lysates from Colo320, SW480, and SW620 CRC cell lines, respectively (71). SW480 lysates were the most effective in stimulating DC maturation and resultingly enhancing T-cell function among the three cell lines. Thus, SW480 lysates were the most efficient in promoting autogenous T-cell-mediated antitumor immune responses (71). The limited therapeutic effect was mainly due to the low immunogenicity of TAAs, so the α-gal epitope was synthesized on the SW620 to increase TAA immunogenicity (72). The α-gal epitope is absent in humans, but natural anti-gal antibody presents in human serum in a large number. DCs were then loaded with the α-gal-expressing SW620 lysate, which stimulated CTL response and increased the frequency of natural killer T cells and CD8^+^ CTLs. Importantly, the CTLs had increased cytotoxicity against tumor cells. Thus, this novel strategy might be an effective treatment approach for CRC patients (72).

Anterior gradient-2 (AGR2) promotes tumor growth, cell migration, and cellular transformation. GM/IL-4DCs were transduced with a recombinant Ad bearing the AGR2 gene (AdAGR2/DCs), which expressed AGR2 protein without any significant changes in DC viability and cytokine secretion compared with unmodified DCs (73). AdAGR2 transduction promoted DC maturation. More importantly, AdAGR2/DCs induced potent AGR2-specific CTLs that could kill AGR2-expressing CRC cell lines. The data indicated that AGR2 might be a potentially promising antigen target for DC-based vaccines against CRC (73).

Several TAA epitopes are used in DC-based vaccines against CRC. Kulikova et al. described an approach to induce an anti-tumor immunity in mononuclear cell (MNC) cultures from CRC patients using DNA-transfected DCs encoding the TAA epitopes of CEA, mucin 4, and the epithelial cell adhesion molecule (74). DCs loaded with polyepitope promoted MNC anti-tumor activity, tumor cell apoptosis, and the frequency of perforin^+^ lymphocytes. Moreover, DCs loaded with polyepitope induced a CTL response that was as efficient by tumor lysate-loaded DCs. Collectively, the findings indicated that polyepitope DNA-transfected DCs were efficient at inducing an antitumor immune response (74). Thus, the DNA construct was likely to be used in DC-based vaccines against CRC.

The optimization of antigen loading is one of the strategies for enhancing the efficacy of DC-based vaccines. CRC patients with liver metastases were vaccinated with either CEA-derived peptide-loaded or CEA mRNA-transfected DCs prior to the surgical resection of the metastases in one clinical trial (75). Approximately 8 out of 11 patients were detected with CEA peptide-specific T cells in the peptide group but none out of 5 patients in the RNA group. This finding indicated that CEA mRNA transfection was not superior to CEA-peptide loading in the generation of tumor-specific immune responses in CRC patients (75).

In a clinical phase I study, patients with advanced CRC received CEA-pulsed DCs mixed with tetanus toxoid and subsequent IL-2 treatment (76). Twelve patients were recruited. There were no severe adverse effects related with the treatment in patients who received the regular 4 DC vaccine injections. Two patients underwent SD, and 10 patients showed disease progression. Approximately 2 out of 9 patients showed an increase in the proliferation of CEA-specific T cells. The CEA-specific immune response was enhanced, and a small fraction of patients benefited from the treatment (76). Thus, these data indicated that it was safe and feasible to treat CRC patients using this strategy. This treatment protocol warranted further assessment in a large cohort of CRC patients.

The monoclonal antibodies against PD-L1/PD-1 have been exploited for the clinical treatment of various tumor types with a favorable therapeutic effect. Hu et al. showed that anti-PD-L1 treatment promoted DC maturation and enhanced the functionality of the mDC1 (77). In addition, anti-PD-L1 treatment might also enhance the number of CTLs with more potent anti-tumor capacity (77). Thus, the combination therapy of DC-based vaccines and anti-PD-L1 was likely to be an effective treatment regimen for CRC patients.

The activation of CD40/CD40L can improve DC-based vaccines against GI cancer, including CRC. Human DCs were loaded with tumor cell lysates followed by the transduction of Ad-carrying human CD40L (Ad-hCD40L) (78). Ad-hCD40L transduction induced a high expression of soluble CD40L and membrane-bound CD40L in/on DCs, which elicited a potent cellular CD40/CD40L interaction among DCs, resulting in the formation of cell aggregates. Thus, the endogenous expression of membrane-bound CD40L and the stimulation of CD40L/CD40 provoked a cellular interaction, which increased the DC function. Importantly, a Th1 cytokine/chemokine expression was induced, enabling the cytotoxicity of effector cells toward the human bile duct and colorectal and pancreatic tumor cells (78). The findings indicate a promising approach for the DC-based immunotherapy of GI malignancies by activating the CD40/CD40L signaling.

### Advances in Dendritic Cell-Based Therapy in Colorectal Cancer

Several meta-analyses of clinical trials with CRC patients all showed that the combination of CIK/DC-CIK immunotherapy and chemotherapy prolonged the survival time, enhanced immune responses, and alleviated chemotherapy-mediated side effects (79–82).

## Perspective

**Figure 1:**
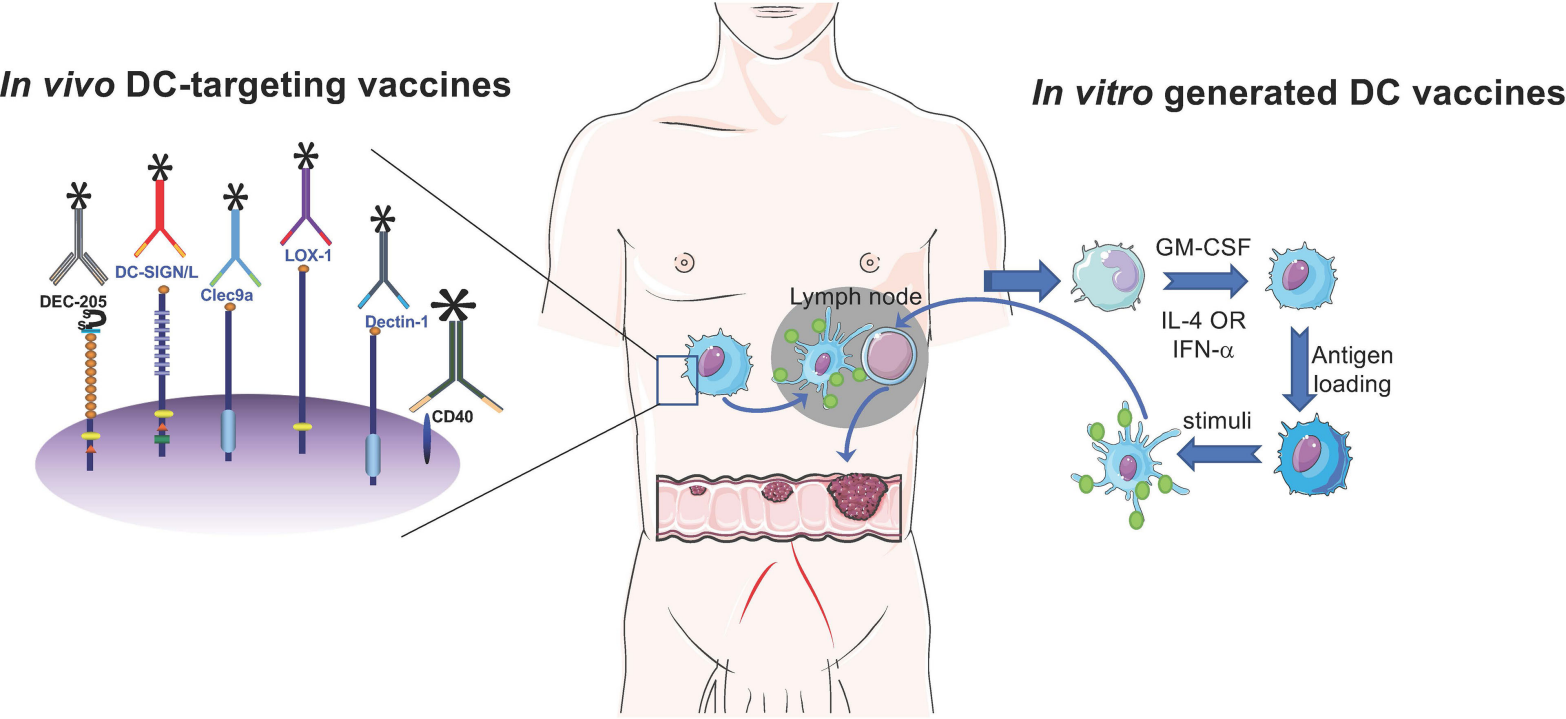
Schematic overview of DC-based CRC vaccines. DC-based vaccines consist of two main approaches: *in vitro* generated DC vaccines and *in vivo* DC-targeting vaccines. *In vivo* DC targeting is a vaccine approach to deliver antigens directly to DCs *in vivo* using chimeric targets composed of an anti-DC receptor antibody and an antigen. *In vitro*-generated vaccines often used monocyte-derived DCs. Briefly, DCs are generated by culture of monocytes in the presence of GM-CSF and IL-4 (or IFN-α), which are then loaded with tumor-associated antigens (TAAs). After maturation, TAA-loaded DCs are injected into CRC patients.

This paper summarized DC subsets in peripheral blood as well as in the tumor from GI cancer patients (**Table 1**). Overall, DC-based vaccines consist of two main approaches: *in vitro* generated DC vaccines and *in vivo* DC-targeting vaccines (**Figure 1**). DC-based vaccines in this review paper were all *in vitro-*generated DC ones. *In vivo* DC targeting is a vaccine approach to deliver antigens directly to DCs *in vivo* using chimeric targets composed of an anti-DC receptor antibody and antigen, which were first studied by Michael Nussenzweig and Ralph Steinman (83, 84). By the immunization of the antigen linked to the anti-DEC-205 antibody, strong, potent, and broader immune responses at low antigen doses were induced in mice. However, in the context of GI cancer patients, DCs *in vivo* demonstrated a functional defect, including DC recruitment, maturation, and cytokine release, which might contribute to tumor growth and progression. Therefore, the use of *in vitro*-generated DC vaccines might be a better option. The clinical trials for the treatment of GI cancer are basically exploiting *in vitro*-generated DCs (**Table 2**).

**Table 1:** Summary of DC subsets and TAAs in GI cancer.

Tumor types	Blood	Tumor mass	Sources of TAAs for DC vaccines	DC-based therapy
EC	/	CD1a^+^ DCs↑ DC-Lamp^+^ DCs↓	EC cell line, 18E7, MAGE-A3	DC vaccine, chemotherapy plus DC injection, DC-CIK
GC	mDC2↑ pDCs↑	CD83^+^ DCs↓ pDCs↑	CSCs, GC cell line	DC vaccine, chemotherapy plus CB-DC-CIK
HCC	CD14^+^ DCs↑ PD-1^+^ mDCs↑ PD-1^+^ pDCs↑	CD83^+^CCR7^-^ DCs↑ pDCs↑	HCC cell line, AFP, DC-derived exosomes, HSP70	DC vaccine, DC-CIK, DC-CIK plus TACE, OK-432-DCs plus TAE, DC-CIK plus anti-PD-1
PC	mDCs↓ pDCs↓	/	PC cell line, primary tumor cells, CSCs, WT1, MSLN, mucin 1, mucin 4, survivin, pBSDL-J28, hTERT, CEA	DC vaccine, DC-CIK, Zol-DCs, and gemcitabine and T cells
CRC	mDCs→ CD85K^+^ mDCs↑ pDCs↓	CD1a^+^/DC-LAMP^+^↑ pDCs↓ mDCs↓	CRC cell lines, α-gal epitope-expressing CRC cell line, AGR2, CEA, mucin 4, epithelial cell adhesion molecule	DC vaccine, DC-CIK

EC, esophageal cancer; GC, gastric cancer; CRC, colorectal cancer; PC, pancreatic cancer; HCC, hepatocellular carcinoma; DC, dendritic cell; mDC, myeloid dendritic cell; pDC, plasmacytoid dendritic cell; AFP, alpha-fetoprotein; HSP70, heat shock protein 70; CSC, cancer stem cell; CIK, cytokine-induced killer cell; TACE, transcatheter arterial chemoembolization; TAE, hepatic arterial embolization; Zol-DC, zoledronate-pulsed DC; AGR2, anterior gradient-2; CEA, carcinoembryonic antigen. ↓decrease; ↑increase.

**Table 2:** Summary of clinical trials of DC-based therapy.

ClinicalTrials.gov Identifier	Status	Diseases	Interventions	Phase
**NCT00005956**	Completed	Breast cancer, GC, ovarian cancer	HER-2/neu intracellular domain protein plus autologous DCs	NA
**NCT00019591**	Completed	CRC	Ras peptide-pulsed DCs	1 and 2
**NCT00027534**	Unknown	Breast cancer, GI cancer, ovarian cancer	Autologous DCs infected with CEA-6D expressing fowlpox-tricom	1
**NCT00103142**	Completed	CRC	Autologous DCs	2
**NCT00154713**	Unknown	CRC	CEA-pulsed DCs	1 and 2
**NCT00176761**	Terminated	CRC	Tumor-pulsed DCs	2
**NCT00228189**	Completed	CRC	CEA-loaded DC vaccine	2
**NCT00311272**	Completed	CRC	DCs loaded with tumor antigens	2
**NCT00558051**	Completed	Metastatic CRC	Alpha-type-1 DC-based vaccines	1
**NCT01413295**	Completed	CRC	Autologous DCs pulsed with tumor antigens	2
**NCT01637805**	Unknown	Stage IV GC	CEA-loaded DC vaccine	1
**NCT01671592**	Completed	CRC	Alpha-type-1 DC vaccines	1
**NCT01691625**	Unknown	EC	Concurrent chemoradiotherapy plus DC-CIK immunotherapy	NA
**NCT01783951**	Completed	GC	DC-CIK	1 and 2
**NCT01839539**	Unknown	CRC	DC-CIK	2
**NCT01885702**	Active, not recruiting	CRC	Neoantigen-loaded DC	1 and 2
**NCT02202928**	Unknown	CRC	Autologous tumor lysate-pulsed DCs and CIK	2
**NCT02215837**	Unknown	GC	Autologous tumor lysate-pulsed DCs plus CIK	2
**NCT02496273**	Active, not recruiting	GC	CEA-loaded DC vaccine	1
**NCT02503150**	Unknown	Metastatic CRC	Autologous tumor lysate- pulsed human DC vaccine	3
**NCT02504229**	Unknown	GC	DC-CIK	2
**NCT02602249**	Unknown	GC	Mucin1-gene-DC-CTL or MUC1-peptide-DC-CTL	1
**NCT02686944**	Completed	GI	Allogeneic, proinflammatory DC suspension	1
**NCT02693236**	Unknown	EC	Mucin1 and survivin-loaded DC plus CIK	1 and 2
**NCT02882659**	Unknown	HCC, CRC	Autologous dendritic killer cell-based immunotherapy	1
**NCT02919644**	Recruiting	Stage IV CRC	Autologous DCs loaded with tumor homogenate	2
**NCT03152565**	Completed	CRC	Avelumab plus autologous DC vaccine	1
**NCT03185429**	Unknown	GI	Tumor-specific antigen-loaded DCs	NA
**NCT03214939**	Unknown	CRC	Autologous antigen-activated DCs	1
**NCT03300843**	Terminated	Melanoma, GI, breast cancer, ovarian cancer,	Peptide-loaded DC vaccine	2
**NCT03410732**	Unknown	GC	Activated autologous DCs	2
**NCT03730948**	Recruiting	CRC	mDC3 vaccine	1
**NCT04567069**	Recruiting	GC	Autologous DCs loaded with MG-7 antigen	1 and 2

EC, esophageal cancer; GI, gastrointestinal cancer; GC, gastric cancer; CRC, colorectal cancer; HCC, hepatocellular carcinoma; NA, not applicable; DC, dendritic cell; CEA, carcinoembryonic antigen; CIK, cytokine-induced killer cell.

The efficacious vaccines should consider several factors, such as DC subsets, tumor-associated antigen targets, antigen-loading methods, proper adjuvants, the selection of various injection routes, and so on. Appropriate adjuvants can dramatically improve the anti-tumoral efficacy of DC-based vaccines. DCIR-targeted vaccines and CD40L (adjuvant) activated DCs, which, in turn, primed CD8^+^ T cells to secrete type 2 cytokines and IFN-γ (85). In contrast, CD8^+^ T cells primed by DCs that were activated with the DCIR-targeted vaccines and TLR7/8 ligands produced higher levels of IFN-γ and granzyme B and perforin but no type 2 cytokines (85). TLR2 ligands are known to promote the induction of Treg cells, which are not likely to be used as adjuvants in the context of cancer (86). The TLR3 ligand induces type 1 IFN responses and promotes cross-presentation (87, 88), which is widely used in clinical trials.

Currently, the whole tumor cell lysates (primary or cell line) are often used for preparing DC-based vaccines in GI cancer, since they comprise whole tumor-associated antigens. The identification of tumor-specific or associated neoantigens is also a key factor that can specifically enhance the efficacy of DC-based vaccines. For DC-based cancer immunotherapy in the future, a combination of existing treatment regimens will be the trend. DC-based vaccines can be combined with chemotherapy that kills tumor cells and release neoantigens. In addition, the combination of DC vaccines and immune checkpoint blockade (ICB) is another trend since the ICB therapy, such as PD-1/PD-L1 inhibitors, enhances the T-cell-mediated immune response. Although the clinical trials indicated that DC-based immunotherapy alone and in combination with chemotherapy was well tolerated and prolonged the survival time of GI cancer patients, the efficacies are still a big challenge.

## Author Contributions

The author confirms being the sole contributor of this work and has approved it for publication.

## Funding

This work was supported by grants from National Key Research and Development Program of China (2021YFC2302403), Tsinghua University Spring Breeze Fund (2020Z99CFG008) and National Natural Science Foundation of China (NSFC) (31991173 and 31991170).

## Conflict of Interest

The author declares that the research was conducted in the absence of any commercial or financial relationships that could be construed as a potential conflict of interest.

## Publisher’s Note

All claims expressed in this article are solely those of the authors and do not necessarily represent those of their affiliated organizations, or those of the publisher, the editors and the reviewers. Any product that may be evaluated in this article, or claim that may be made by its manufacturer, is not guaranteed or endorsed by the publisher.

## Glossary

**Table:**

ICI	immune checkpoint inhibitor
GI	gastrointestinal
EC	esophageal cancer
GC	gastric cancer
CRC	colorectal cancer
PC	pancreatic cancer
HCC	hepatocellular carcinoma
DC	dendritic cell
CTL	cytotoxic T lymphocyte
iDC	immature dendritic cell
CD8^+^ TILs	tumor-infiltrating CD8^+^ T cells
TAA	tumor-associated antigen
MAGE-A3	melanoma-associated antigen 3
HPV	human papillomavirus
CALR	calreticulin
MHC	major histocompatibility
Ad	adenovirus
CIK	cytokine-induced killer cells
Tregs	regulatory T cells
pDC	plasmacytoid dendritic cell
TME	tumor microenvironment
mDC2	CD1c^+^ myeloid dendritic cell
CSC	cancer stem cell
SLC	secondary lymphoid tissue chemokine
CB-DC-CIK	cord blood-derived dendritic cell and cytokine-induced killer cell
DEG	differentially expressed gene
OS	overall survival
PBMC	peripheral blood mononuclear cell
IDO	indoleamine-2,3-dioxygenase
mDC1	CD141^+^ mDC
LAG-3	lymphocyte activation gene 3
LXR	liver X receptor
AFP	alpha-fetoprotein
nAFP	normal AFP
tAFP	tumor-derived AFP
LMM	low molecular mass
rAAV	recombinant adeno-associated virus
DEXs	DC-derived exosomes
HSP70	heat shock protein 70
DFS	disease-free survival
ATVAC	autologous tumor lysate-pulsed DC vaccine plus *ex vivo* activated T-cell transfer
Nb	nanobody
FC	tumor-specific DC/tumor-fusion cell
PCNA	proliferating cell nuclear antigen
LCSC	liver cancer stem cell
TACE	transcatheter arterial chemoembolization
TAE	hepatic arterial embolization
PDAC	pancreatic ductal adenocarcinoma
LPS	lipopolysaccharide
BxCM	BxPC-3-culture medium
TDX	tumor-derived exosome
RFXAP	regulatory factor X-associated protein
TFF2	trefoil factor 2
Reg3A	regenerating islet-derived protein 3a
WT1	Wilms’ tumor 1
MSLN	mesothelin
FAPP	fetoacinar pancreatic protein
hTERT	human telomerase reverse transcriptase
CEA	carcinoembryonic antigen
PEU-FNI	preoperative endoscopic ultrasound-guided fine-needle injection
Zol-DCs	zoledronate-pulsed DCs
SD	stable disease
NLR	neutrophil/lymphocyte ratio
AGR2	anterior gradient-2
MNC	mononuclear cell
Ad-hCD40L	Ad-encoding human CD40L
